# Spirometric changes during exacerbations of COPD: a post hoc analysis of the WISDOM trial

**DOI:** 10.1186/s12931-018-0944-3

**Published:** 2018-12-13

**Authors:** Henrik Watz, Kay Tetzlaff, Helgo Magnussen, Achim Mueller, Roberto Rodriguez-Roisin, Emiel F. M. Wouters, Claus Vogelmeier, Peter M. A. Calverley

**Affiliations:** 1 Pulmonary Research Institute at Lungen Clinic Grosshansdorf, Airway Research Center North (ARCN), German Center for Lung Research (DZL), Wöhrendamm 80, 22927 Grosshansdorf, Germany; 2 Boehringer Ingelheim International GmbH, Ingelheim am Rhein, Germany; 3 Department of Sports Medicine, University of Tübingen, Tübingen, Germany; 4 Boehringer Ingelheim Pharma GmbH & Co. KG, Biberach an der Riss, Germany; 5 Hospital Clínic IDIBAPS-CIBERES, Universitat de Barcelona, Barcelona, Spain; 6 Department of Respiratory Medicine, Maastricht University Medical Center, Maastricht, Netherlands; 7 Department of Medicine, Pulmonary and Critical Care Medicine, University Medical Center Giessen and Marburg, Philipps-Universität Marburg, Member of the German Center for Lung Research (DZL), Marburg, Germany; 8 Institute of Ageing and Chronic Disease, Clinical Science Centre, University Hospital Aintree, Liverpool, UK

**Keywords:** COPD, COPD exacerbation, Lung function, FEV_1_, Home-based spirometry

## Abstract

**Background:**

Exacerbations of chronic obstructive pulmonary disease (COPD) are associated with loss of lung function and poor outcomes for patients. However, there are limited data on the time course of changes in forced expiratory volume in 1 s (FEV_1_) preceding the first reported symptom and after the start of an exacerbation.

**Methods:**

WISDOM was a multinational, randomized, double-blind, active-controlled, 52-week study in patients with severe-to-very severe COPD. Patients received triple therapy (long-acting muscarinic antagonist and long-acting β_2_-agonist/inhaled corticosteroid [ICS]) for 6 weeks, and were randomized to continue triple therapy or stepwise withdrawal of the ICS (dual bronchodilator group). After suitable training, patients performed daily spirometry at home using a portable, battery-operated spirometer. In the present post hoc analysis, patients who continued to perform daily home spirometry and completed at least one measurement per week for a 56-day period before and after the start of a moderate or severe exacerbation were included. Missing values were imputed by linear interpolation (intermittent), backfilling (beginning) or carry forward (end). Exacerbation onset was the first day of a reported symptom of exacerbation.

**Results:**

Eight hundred and eighty-eight patients in the WISDOM study had a moderate/severe exacerbation after the complete ICS withdrawal visit; 360 of them contributed at least one FEV_1_ measure per week for the 8 weeks before and after the event and are included in this analysis.

Mean daily FEV_1_ began to decline from approximately 2 weeks before the onset of symptoms of an exacerbation, dropping from 0.907 L (mean Days − 56 to − 36 before the exacerbation) to 0.860 L on the first day of the exacerbation. After the exacerbation, mean FEV_1_ improved but did not return to pre-exacerbation levels (mean Days 36–56 after the exacerbation, 0.875 L).

The pattern of FEV_1_ changes around exacerbations was similar in the triple therapy and dual bronchodilator groups, and a similar pattern was seen in moderate and severe exacerbations when analysed separately.

**Conclusions:**

Mean lung function starts to decline prior to the first reported symptoms of an exacerbation, and does not recover to pre-exacerbation levels 8 weeks after the event.

**Trial registration:**

WISDOM (ClinicalTrials.gov number, NCT00975195).

**Electronic supplementary material:**

The online version of this article (10.1186/s12931-018-0944-3) contains supplementary material, which is available to authorized users.

## Background

Exacerbations of chronic obstructive pulmonary disease (COPD) are associated with both short-term loss and long-term decline of lung function [1–3]. Previous studies have shown that lung function drops at the time of an exacerbation and does not always fully recover to pre-exacerbation levels [4, 5]. Much of what is known about the impact of exacerbations on lung function is from analysing long-term lung function changes in patients who experienced exacerbations over the course of a clinical trial, with lung function measured at scheduled in-clinic visits rather than at the time of the event [2, 3].

Some studies have measured lung function at the time of and immediately after an event [6, 7]. However, there are limited data available on the time course of lung function changes preceding exacerbations and immediately following exacerbations. Seemungal et al. [4] showed that peak expiratory flow rate (PEFR) remains relatively stable before the onset of an exacerbation, while symptoms already deteriorate. By contrast, Calverley et al. [8] observed a decrease of around 8% in peak expiratory flow (PEF) in the 2–3 weeks before an exacerbation.

There remains a need to better understand the spirometric changes around the time of an exacerbation, as it is currently difficult to identify exacerbations early. This is of clinical relevance as treating exacerbations earlier is associated with faster recovery and reduced risk of hospitalization [9].

The WISDOM study was a 12-month inhaled corticosteroid (ICS) withdrawal study in patients with severe-to-very severe COPD. It showed that there was no increased risk of exacerbations following stepwise withdrawal of fluticasone propionate in patients receiving tiotropium and salmeterol [10]. In the WISDOM study, following suitable training, patients performed daily spirometry at home, providing an opportunity to examine the changes in forced expiratory volume in 1 s (FEV_1_) that occur prior to and immediately after an exacerbation.

The aim of the present post hoc analysis was to characterize the lung function profile of patients prior to, during and following the first moderate/severe and severe exacerbation during the WISDOM trial.

## Methods

### Study design

The WISDOM study methodology has been published previously [10, 11]. In brief, this was a multinational, randomized, double-blind, parallel-group, active-control study. Patients entered a 6-week triple-therapy run-in with long-acting muscarinic antagonist (LAMA) and long-acting β_2_-agonist (LABA)/ICS (tiotropium and salmeterol/fluticasone propionate). Patients were then randomized (study Week 0) to either continue triple therapy for 52 weeks or to continue receiving salmeterol and tiotropium (dual bronchodilator therapy) whilst discontinuing ICS in a stepwise manner over 12 weeks. ICS were completely discontinued in this group at Week 12. The primary endpoint was the time to the first moderate or severe COPD exacerbation during the 12-month study period. These results and further post hoc analyses have been published previously [10, 12, 13]. The study was performed in accordance with the Declaration of Helsinki, the International Conference on Harmonisation’s Harmonised Tripartite Guideline for Good Clinical Practice and local regulations. The protocol was approved by the ethics research board of the respective institutions, and all patients provided written informed consent.

### Patients and treatments

Patients were ≥ 40 years of age, were either current or former smokers, had been diagnosed with severe or very severe COPD (defined as an FEV_1_ < 50% of the predicted volume and < 70% of the forced vital capacity after bronchodilation), and a history of at least one documented exacerbation in the 12 months prior to screening. Further inclusion and exclusion criteria have been reported elsewhere [11].

Patients were randomized to continue receiving tiotropium, salmeterol and fluticasone propionate, or to have fluticasone propionate withdrawn over a 12-week period (further details are given in the Supplementary Methods in the Additional file 1).

### Exacerbations

Patients completed a simple daily paper diary, recording changes in respiratory symptoms and the use of medications between visits. Diaries were used to help patients report symptoms and medication changes to investigators. Moderate exacerbations were defined as an increase of at least two lower respiratory tract symptoms related to COPD (shortness of breath, sputum production [volume], sputum purulence, cough, wheezing or chest tightness), or the new onset of two or more such symptoms, with at least one symptom lasting 3 or more days and for which antibiotics, systemic glucocorticoids or both were prescribed. A severe exacerbation was defined as an exacerbation requiring hospitalization. The start date of an exacerbation was defined as the date of onset of the first COPD symptom that was part of the exacerbation and was determined by the investigator reviewing the diary cards. The end of the exacerbation was determined by the investigator reviewing the diary cards and stopping exacerbation treatment.

In the case of an exacerbation, patients could be treated with oral glucocorticoids, and/or antibiotics and further treatments as deemed medically necessary.

### Lung function

Following suitable training, patients performed daily spirometry throughout the 52-week study. Home-based measurements were performed by patients each morning before administration of the study drug using a portable, battery-operated, ultrasound, transit-time-based electronic spirometer (EasyOne® NDD Medical Technologies, Chelmsford, MA, USA, and Zurich, Switzerland). Patients performed at least three efforts each day, and the highest FEV_1_ was selected for analysis [14]. Automatic feedback was provided to the patient if effort was not in line with American Thoracic Society (ATS)/European Respiratory Society (ERS) criteria [15]. Data were retrieved at clinic visits, and a pulmonologist performed a central over-read of all lung function data and graded it for acceptability according to ATS/ERS criteria. All spirometric techniques and equipment used were in accordance with ATS/ERS recommendations [15].

We have previously shown that home-based FEV_1_ is a reliable measure, and there was a good agreement between the home-based spirometry results and the in-clinic spirometry in the WISDOM trial. However, the home-based measures of FEV_1_ were consistently approximately 50 mL lower than in-clinic values [14].

### Statistical analysis

For the present post hoc analysis, we calculated absolute and percentage change from pre-exacerbation period baseline (mean of the period 6–8 weeks before the exacerbation) in on-treatment daily FEV_1_ for 8 weeks before and after the first day of the first moderate/severe on-treatment COPD exacerbation, as well as change in on-treatment daily FEV_1_ in the subsets of moderate and severe on-treatment COPD exacerbations. We also calculated weekly means for FEV_1_ and the change from Week − 8.

No inferential statistical analyses were conducted. Descriptive statistics were calculated for demographic variables. Data for change in on-treatment daily and weekly FEV_1_ up to and following the first day of the first exacerbation (moderate, moderate/severe and severe) were plotted graphically for visual interpretation. For this analysis, patients who had at least one measurement per week in a 56-day period before and after the start of a moderate or severe exacerbation were included. Missing values were imputed by linear interpolation (intermittent), backfilling (beginning) or carry forward (end).

The analysis includes patients’ first exacerbation after the ICS withdrawal visit.

## Results

### Patients

Of 2488 patients randomized into the WISDOM study, 888 experienced a moderate/severe exacerbation between Week 12 (the time of complete ICS withdrawal) and Week 52; 182 patients experienced severe exacerbations. Of the patients with a moderate/severe exacerbation, a total of 360 patients completed at least one lung function measurement per week in the 8 weeks prior to and after the exacerbation, so were included in this analysis: 317 with moderate exacerbations and 43 with severe exacerbations. The number of exacerbations occurring during triple therapy was similar to those occurring during dual bronchodilation. The demographics of the patients included in this analysis are shown in Table 1; patients were predominantly male (78.9%) and had a mean age of 63.7 years. Mean in-clinic post-bronchodilator FEV_1_ at screening was 0.923 L (32.7% predicted).

**Table 1:** Descriptive statistics for patients included in this analysis and for all treated patients in the WISDOM population

	Analysis	All treated
Patients, *n*	360	2485
Males, *n* (%)	284 (78.9)	2049 (82.5)
Age, mean years (SD)	63.7 (8.5)	63.8 (8.5)
BMI, mean kg/m^2^ (SD)	25.4 (5.4)	25.2 (5.1)
COPD duration, mean years (SD)	8.7 (6.2)	7.9 (6.2)
Smoking status, *n* (%)		
Former smoker	236 (65.6)	1654 (66.6)
Current smoker	124 (34.4)	831 (33.4)
Smoking history, mean pack-years (SD)	43.0 (21.6)	45.0 (24.3)
In-clinic post-bronchodilator FEV_1_, mean L (SD)	0.923 (0.285)	0.933 (0.297)
In-clinic post-bronchodilator FEV_1_, mean % predicted (SD)	32.7 (8.4)	32.8 (9.1)
Mean home-measured FEV_1_ in the period 6–8 weeks before index exacerbation, L (SD)	0.907 (0.321)	N/A
GOLD status, n (%)		
2	1 (0.3)	1 (0.6)
3	218 (60.6)	101 (58.0)
4	141 (39.2)	72 (41.4)
Number of patients included with a moderate exacerbation, *n* (%)	317 (88.1)	N/A
Number of patients included with a severe exacerbation, n (%)	43 (11.9)	N/A

BMI, body mass index; COPD, chronic obstructive pulmonary disease; FEV_1_, forced expiratory volume in 1 s; GOLD, Global Initiative for Chronic Obstructive Lung Disease; ICS, inhaled corticosteroid; LABA, long-acting β_2_-agonist; LAMA, long-acting muscarinic antagonist; N/A, not applicable; SD, standard deviation

The demographics of the patients who provided FEV_1_ data in this analysis are similar to the demographics of the wider population of patients in WISDOM (Additional file 2: Table S1) [10]. The mean (standard deviation [SD]) duration of moderate exacerbations was 18 (31) days; for severe exacerbations, it was 25 (28) days.

### Daily and weekly FEV_1_ changes before and after exacerbations

**Fig. 1:**
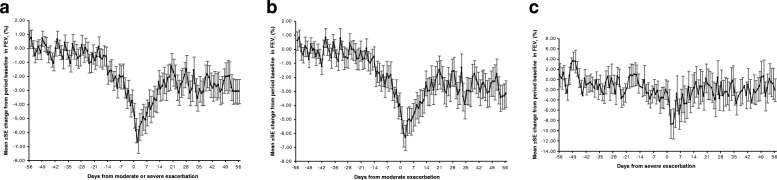
**a** Percentage change from period baseline in on-treatment daily FEV_1_ before and after first moderate/severe exacerbation. **b** Percentage change from period baseline in on-treatment daily FEV_1_ before and after first moderate exacerbation. **c** Percentage change from period baseline in on-treatment daily FEV_1_ before and after first severe exacerbation. All graphs show both treatments combined. Plots include patients whose first exacerbation after the ICS withdrawal visit was neither preceded by an exacerbation of any severity in 8 weeks prior nor followed by a further exacerbation of any severity within 8 weeks (56 days) after exacerbation. Day 0 is the day of the exacerbation. Period baseline is the mean of Days − 56 to − 36. FEV_1_, forced expiratory volume in 1 s; ICS, inhaled corticosteroids; SE, standard error

**Fig. 2:**
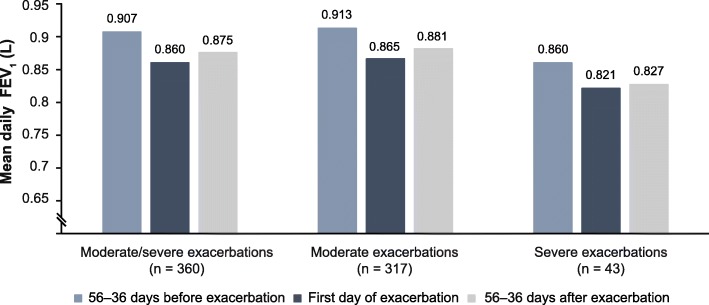
FEV_1_ before, during and after moderate/severe exacerbations, moderate exacerbations and severe exacerbations. FEV_1_, forced expiratory volume in 1 s

For all patients combined (triple therapy and dual bronchodilation; Fig. 1), FEV_1_ was fairly stable from 8 to 3 weeks before the first moderate/severe exacerbation using the average of the stable period Week − 8 to Week − 6 (i.e. Days − 56 to − 36) as baseline. Mean FEV_1_ in this baseline period was 0.907 L. Looking at the change from this baseline revealed a pronounced drop in FEV_1_ that began in the 2 weeks prior to the exacerbation. While the 90% confidence interval (CI) for the change included zero for all days before Day − 14, from Day − 14 onwards the 90% CI was below zero, indicating a drop in FEV_1_. FEV_1_ decreased from − 0.58% (90% CI: -1.67, 0.53; absolute value − 7 mL) on Day − 15 to − 1.91% (90% CI: -2.96, − 0.86; absolute value − 18 mL) on Day − 14, and dropped down to − 2.06% (90% CI: -3.42, − 0.71; absolute value − 21 mL) on Day − 7 and − 4.79% (90% CI: -6.26, − 3.32; absolute value − 47 mL) on Day 0. On Day 2 of the exacerbation, FEV_1_ dropped to its lowest value: − 6.65% (90% CI: -8.06, − 5.23) below the stable period 6–8 weeks before the exacerbation (absolute drop − 69 mL). Following the first day of the exacerbation, FEV_1_ improved over approximately 14 days, after which stable levels were observed. Notably, post-exacerbation lung function did not reach the pre-exacerbation levels (36–56 days after exacerbation, mean FEV_1_ 0.875 L; Fig. 2).

We also examined moderate exacerbations and severe exacerbations separately (Fig. 1). For both exacerbation types, change from baseline in lung function remained stable from 8 to 3 weeks before the first exacerbation. At around 2 weeks before the exacerbation, lung function declined until the exacerbation, and did not recover to pre-exacerbation levels within 8 weeks after the start of the exacerbation (Figs. 1b & c and 2). The magnitude of the mean fall in FEV_1_ before exacerbation to the first day of exacerbation was − 48 mL (− 5.08%), dropping to a low of − 66 mL (− 6.36%) on Day 2 for moderate exacerbations, and − 39 mL (− 2.63%) with a further drop to − 85 mL (− 8.77%) on Day 2 of the exacerbation for severe exacerbations (Figs. 1b & c and 2).

**Fig. 3:**
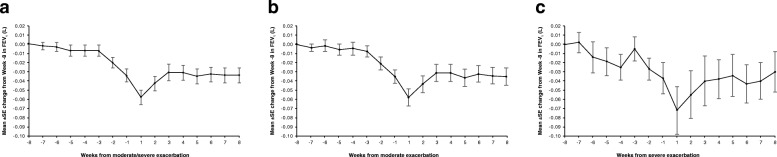
**a** Change from Week − 8 in on-treatment weekly FEV_1_ before and after first moderate/severe exacerbation. **b** Change from Week − 8 in on-treatment weekly FEV_1_ before and after first moderate exacerbation. **c** Change from Week − 8 in on-treatment weekly FEV_1_ before and after first severe exacerbation. All graphs show both treatments combined. Plots include patients whose first exacerbation after the ICS withdrawal visit was neither preceded by an exacerbation of any severity in 8 weeks prior nor followed by a further exacerbation of any severity within 8 weeks (56 days) after exacerbation. Patients had weekly means available for the entire period of interest. FEV_1_, forced expiratory volume in 1 s; ICS, inhaled corticosteroids; SE, standard error

Mean change in on-treatment weekly FEV_1_ before and after the first moderate/severe, moderate or severe exacerbations, for both treatments combined, followed similar patterns to the daily data (Fig. 3).

### FEV_1_ changes by treatment group

**Fig. 4:**
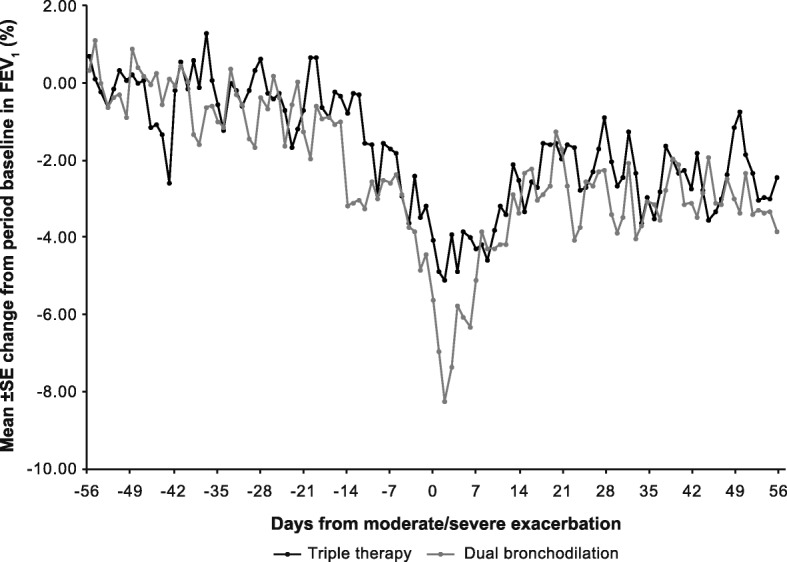
Percentage change from period baseline in on-treatment daily FEV_1_ before and after first exacerbation by treatment group. Plot includes patients whose first moderate or severe exacerbation after the ICS withdrawal visit was neither preceded by an exacerbation of any severity in 8 weeks prior nor followed by a further exacerbation of any severity within 8 weeks (56 days) after exacerbation. Day 0 is the day of the exacerbation. Period baseline is the mean of Days − 56 to − 36. FEV_1_, forced expiratory volume in 1 s; ICS, inhaled corticosteroids; SE, standard error

For all moderate/severe exacerbations, when the treatment groups were plotted separately (Fig. 4), a similar pattern emerged as for the lung function changes in the combined treatment group (Fig. 1). There was a slightly larger drop in FEV_1_ in the dual bronchodilator group than in the triple therapy arm in the days after the exacerbation, but after 7 days the change in lung function was similar in the two groups.

## Discussion

This is the first study to show a decrease in daily-measured lung function before an exacerbation in a large group of patients with severe COPD and a low FEV_1_ at baseline (33% predicted). This decrease in FEV_1_ was observed from approximately 2 weeks before the start of an exacerbation, and although improvements were seen post-exacerbation, FEV_1_ did not recover to pre-exacerbation levels up to 8 weeks after the start of the exacerbation. The post-exacerbation FEV_1_ levels could either reflect the fact that the drop in FEV_1_ never recovered, did not recover within 8 weeks after the start of the exacerbation, or that there was progressive lung function decline over time. Interestingly, another analysis of a large clinical trial has shown that the rate of lung function decline increases following a single exacerbation [16].

There are limited data in which the daily lung function around the time of an exacerbation has been studied, though our results are consistent with previous studies that have shown that exacerbations are associated with loss in lung function [1–3]. One other study that did report daily lung function was published by Seemungal et al. [4] – a study in which a cohort of 101 patients recorded daily PEFR measurements over 2.5 years. In their cohort, there was no change in PEFR in the days prior to an exacerbation, unlike the results presented here. The PEFR results did, however, show incomplete recovery of lung function [4], as was observed in this WISDOM analysis. Another study that measured PEF before and after exacerbations showed a small drop in PEF prior to the exacerbations that then returned to pre-exacerbation levels within 2 weeks of the exacerbation; however, increased symptoms were still seen 4–8 weeks after the exacerbation [17].

Interestingly, another small study measured lung function at baseline and during an exacerbation in a severe COPD population similar to ours, and found a 40 mL drop in FEV_1_ at the time of the exacerbation [18], an observation consistent with our results.

The daily lung function data also show that the time course of the changes in lung function around exacerbations was similar in patients who withdrew from ICS to those in patients who continued ICS. This suggests that the objective change associated with an exacerbation occurring during ICS treatment was similar to that experienced when this therapy was not used.

The main limitation of the analysis is that it only included patients who could continue to perform home-based spirometry manoeuvres during and after their exacerbation, and patients with more severe symptomatic exacerbations may be more likely to stop performing the home-based measurements. It is possible that prior to more severe exacerbations the drop in FEV_1_ may be more pronounced. However, the demographics of the patients included in this analysis are similar to the demographics of the wider WISDOM population. Another limitation of this study is the inherent variability in FEV_1_, as the repeatability of the measure in individuals is greater than the differences we observed [19]. This raises questions about the possibility of using the drop in lung function as a predictive measure, but does not contradict our findings about the drop in group mean FEV_1_.

One strength of the analysis is that, unlike in observational data, maintenance therapy was standardized. The WISDOM study offers a unique opportunity to closely study the daily lung function of a relatively large number of patients before, during and after exacerbations of COPD occurring during triple therapy compared with dual bronchodilation.

Identifying exacerbations before they fully develop could improve their treatment. Although blood eosinophil count and exacerbation history can both identify patients at increased risk of exacerbation [13, 20–22], it remains difficult to predict exacerbations of COPD. It is unlikely that all COPD exacerbations could be predicted by a drop in FEV_1_ before the event, but it is possible that there is a subset of exacerbations that could be identified by daily home-based spirometry [14] in patients at high risk of exacerbations. Future work is needed to identify cut-offs and to develop algorithms for prediction of exacerbations.

## Conclusions

Overall, a small loss in lung function is observed in patients with severe COPD in the 2 weeks prior to the onset of symptoms of an exacerbation, and mean FEV_1_ did not fully recover 8 weeks after the start of the exacerbation.

## Additional files

Additional file 1: Supplementary Methods. (DOCX 33 kb)

Additional file 2: **Table S1**. Baseline characteristics by treatment group for patients included in this analysis and for all treated patients in the WISDOM population. (DOCX 36 kb)

## Abbreviations

ATS: American Thoracic Society
CI: Confidence interval
COPD: Chronic obstructive pulmonary disease
ERS: European Respiratory Society
FEV_1_: Forced expiratory volume in 1 s
ICS: Inhaled corticosteroids
LABA: Long-acting β_2_-agonist
LAMA: Long-acting muscarinic antagonist
PEF: Peak expiratory flow
PEFR: Peak expiratory flow rate
SD: Standard deviation
