# Supplementary material for: Spirometric changes during exacerbations of COPD: a post hoc analysis of the WISDOM trial
Source: Respir Res. 2018 Dec 13;19:251. doi: 10.1186/s12931-018-0944-3 (PMC6293570; doi:10.1186/s12931-018-0944-3)
Supplement: Supplementary file 1 — Supplementary Methods. (DOCX 33 kb) [file 12931_2018_944_MOESM1_ESM.docx]

**ADDITIONAL FILE**

**Spirometric changes during exacerbations of COPD: a post hoc analysis of the
WISDOM trial**

Henrik Watz, Kay Tetzlaff, Helgo Magnussen, Achim Mueller, Roberto Rodriguez-Roisin, Emiel FM Wouters, Claus Vogelmeier, Peter MA Calverley

**SUPPLEMENTARY METHODS**

**Treatments**

During the run-in period, patients were treated with tiotropium 18 µg once daily (via the HandiHaler^®^), salmeterol xinafoate 50 µg twice daily and fluticasone propionate 500 µg twice daily (each via a metered-dose inhaler). These dosages were maintained throughout the randomized phase for those continuing triple therapy. For those in the inhaled corticosteroid (ICS) withdrawal (dual bronchodilation) group, the total daily fluticasone dose was reduced every 6 weeks in the following stepwise increments: 1000 μg to 500 μg (Week 0); then to 200 μg (Week 6); and finally to 0 μg (placebo) (Week 12). From Week 18, patients were considered to be undergoing stable treatment, as by this time patients in the ICS withdrawal group would have had 6 weeks of no ICS treatment.
