# Supplementary material for: Spirometric changes during exacerbations of COPD: a post hoc analysis of the WISDOM trial
Source: Respir Res. 2018 Dec 13;19:251. doi: 10.1186/s12931-018-0944-3 (PMC6293570; doi:10.1186/s12931-018-0944-3)
Supplement: Supplementary file 2 — Table S1. Baseline characteristics by treatment group for patients included in this analysis and for all treated patients in the WISDOM population. (DOCX 36 kb) [file 12931_2018_944_MOESM2_ESM.docx]

**ADDITIONAL FILE**

**Spirometric changes during exacerbations of COPD: a post hoc analysis of the
WISDOM trial**

Henrik Watz, Kay Tetzlaff, Helgo Magnussen, Achim Mueller, Roberto Rodriguez-Roisin, Emiel FM Wouters, Claus Vogelmeier, Peter MA Calverley

**SUPPLEMENTARY TABLES**

**Table S1.** Baseline characteristics by treatment group for patients included in this analysis and for all treated patients in the WISDOM population.

|  | **Analysis** | | **All treated** | |
| --- | --- | --- | --- | --- |
|  | **Triple therapy (LAMA and LABA/ICS)** | **Dual bronchodilation (LAMA and LABA)** | **Triple therapy (LAMA and LABA/ICS)** | **Dual bronchodilation (LAMA and LABA)** |
| Patients, *n* | 174 | 186 | 1243 | 1242 |
| Males, *n* (%) | 140 (80.5) | 144 (77.4) | 1013 (81.5) | 1036 (83.4) |
| Age, mean years (SD) | 63.5 (9.0) | 63.9 (8.1) | 63.6 (8.6) | 64.0 (8.4) |
| BMI, mean kg/m^2^ (SD) | 25.4 (5.7) | 25.3 (5.2) | 25.3 (5.1) | 25.2 (5.1) |
| COPD duration, mean years (SD) | 8.1 (5.9) | 9.3 (6.5) | 7.8 (6.0) | 8.0 (6.5) |
| Smoking status, *n* (%) |  |  |  |  |
| Former smoker | 108 (62.1) | 128 (68.8) | 811 (65.2) | 843 (67.9) |
| Current smoker | 66 (37.9) | 58 (31.2) | 432 (34.8) | 399 (32.1) |
| Smoking history, mean pack-years (SD) | 42.9 (20.6) | 43.1 (22.5) | 44.8 (23.7) | 45.3 (24.8) |
| Screening lung function |  |  |  |  |
| Post-bronchodilator FEV_1_, mean L (SD) | 0.923 (0.274) | 0.923 (0.295) | 0.929 (0.290) | 0.938 (0.304) |
| Post-bronchodilator FEV_1_ mean % predicted (SD) | 32.7 (8.5) | 32.7 (8.4) | 32.7 (8.9) | 32.8 (9.2) |
| GOLD status, n (%)  1  2  3  4 | 0 (0.0)  1 (0.6)  101 (58.0)  72 (41.4) | 0 (0.0)  0 (0.0)  117 (62.9)  69 (37.1) | 1 (0.1)  6 (0.5)  760 (61.1)  473 (38.1) | 2 (0.2)  3 (0.2)  761 (61.3)  474 (38.2) |
| Number of patients included with a moderate exacerbation, *n* (%) | 155 (89.1) | 162 (87.1) | N/A | N/A |
| Number of patients included with a severe exacerbation, n (%) | 19 (10.9) | 24 (12.9) | N/A | N/A |

BMI, body mass index; COPD, chronic obstructive pulmonary disease; FEV_1_, forced expiratory volume in 1 second; GOLD, Global Initiative for Chronic Obstructive Lung Disease; ICS, inhaled corticosteroids; LABA, long-acting β_2_-agonist; LAMA, long-acting muscarinic antagonist; SD, standard deviation.
